# Global, regional and national patterns and gender disparity of intraocular foreign bodies from 1990 to 2021

**DOI:** 10.3389/fpubh.2025.1620358

**Published:** 2025-06-25

**Authors:** Yi Shan, Fei Chen, Dong Xu, Wenqing Jia, Shangchen Hao

**Affiliations:** Department of Ophthalmology, The First Affiliated Hospital of Zhengzhou University, Zhengzhou, China

**Keywords:** intraocular foreign bodies, global burden of disease, prevalence, disability-adjusted life years, sociodemographic index

## Abstract

**Purpose:**

To describe and evaluate the gender and socioeconomic disparities in the global burden of intraocular foreign bodies (IOFBs) from 1990 to 2021.

**Methods:**

Gender-specific prevalence and disability-adjusted life years (DALYs) of IOFBs by year, age, geography and socioeconomic status were extracted from the Global Burden of Disease Study 2021. We used the Wilcoxon signed rank test and linear regression analysis to research the relationship between the age-standardized DALYs rate and gender difference (males minus females) and Socio-demographic Index (SDI).

**Results:**

The total all-age prevalence and DALYs due to IOFBs rose by 41.9 and 35.5%, respectively, from 1990 to 2021, and the age-standardized prevalence and DALYs rates decreased by 15.2 and 19.6%. The IOFBs burden was greater among middle-aged and older adult men, especially in 45–49 years. The burden of IOFBs was concentrated in countries of Western Europe, East Asia, High-income North America and Southern Latin America. The age-standardized DALYs rates of males were significantly higher than those of females in all five SDI groups (*p* < 0.001) in 2021. Pearson’s correlations (r = 0.3093, *p* < 0.001) and linear regression (Y = 4.850*X − 1.857) revealed a significant positive association between gender differences and SDI. The increase in the all-age DALYs of IOFBs was lowest compared with other eye diseases in GBD 2021. The IOFBs had the greatest gender-related differences compared with other eye disorders.

**Conclusion:**

The burden of IOFBs is higher among men in terms of age, region, and SDI categories. Male workers in regions with higher SDI should receive more attention. Measures are needed to improve eye protection and reduce eye injuries among males in the workplace.

## 1 Introduction

Open globe injury (OGI) is a common and serious eye disease, which can lead to irreversible vision impairment and low quality of life (1, 2). According to data from the World Health Organization (WHO) Blindness Data Bank, approximately 55 million eye injuries are reported worldwide yearly, resulting in 2.3 million cases with low bilateral vision, 19 million with unilateral blindness or low vision, and 750,000 hospitalizations (3). Intraocular foreign bodies (IOFBs), which may be magnetic or non-magnetic and are located in the interior of the eyeball, comprise 18–41% of OGIs (4–6), and 5–31% of patients with IOFBs have a final visual acuity below 3/60 (7–9). Most IOFBs are cause by small projectiles from hammering on metal or stone, machine-tool uses, firing of weapons, explosions, motor vehicle accidents, and lawnmower accidents (10, 11), and the majority are metallic foreign bodies (6). Chemical injury due to metallic foreign bodies can seriously damage eye tissue. The majority of post-traumatic IOFBs are located in the posterior segment, including the vitreous cavity, posterior wall of the eyeball, and the retina (10). Continuous stimulation of the intraocular tissue caused by long-term IOFBs retention may result in a series of complications.

Global disability-adjusted life years (DALYs) due to IOFBs increased by 43.7% from 1990 to 2017, according to the Global Burden of Disease (GBD) study (12). The DALYs due to IOFBs were found predominantly among males from 1990 to 2017, with > 70% of all injuries and > 95% of occupational injuries occurring in men (13–15). Generally, men accounted for 80% of OGIs (almost six times the amount of the women) (15, 16). The higher incidence rate of IOFBs in males was associated with toy guns, ball sports, fights among teenage boys, and accidents and construction work among adult men (14, 15). Disparities in healthcare quality are known to be related to socioeconomic status. The cost of work-related OGIs in the United States has been reported to reach $300 million per year in lost productivity, medical expenses, and workers’ compensation (14).

IOFBs are usually accompanied by poor visual outcomes and high medical expenses (17, 18). Early diagnosis and treatment are effective in preserving the remaining vision and reducing the probability of blindness. However, the systematic analyses of gender and socioeconomic inequality in the health burden of IOFBs have been rare. Understanding gender and socioeconomic patterns of imbalance is essential for developing health policies. This study analyzed the prevalence and DALYs data from the GBD 2021 study by year, age, sex, geography, and socioeconomic status to enhance our understanding of the global burden of IOFBs.

## 2 Methods

### 2.1 Data sources

The GBD 2021 study (19) collected and analyzed data on the incidence and prevalence of 369 diseases and injuries in 204 countries and territories, 21 regions, and seven super-regions. Details on the GBD methodology have been reported previously (19). Prevalence refers to the total number of cases of a given disease in a specified population at a designated time. DALYs refers to the sum of years lost due to premature death and years lived with disability.

We obtained the following data from the Global Health Data Exchange,^1^ including: (1) Global total and gender-specific burden due to IOFBs, containing all-age prevalence and DALYs, age-standardized prevalence and DALYs rate from 1990 to 2021; (2) Global total and gender-specific prevalence and DALYs rate by different age group (< 5 years, 5–9 years, 10–14 years, 15–19 years, 20–24 years, 25–29 years, 30–34 years, 35–39 years, 40–44 years, 45–49 years, 50–54 years, 55–59 years, 60–64 years, 65–69 years, 70–74 years, 75–79 years, 80–84 years, 85–89 years, 90–94 years, ≥ 95 years) in 2021; (3) Gender-specific age-standardized prevalence and DALYs rate in 21 GBD regions in 2021; (4) All-age DALYs number and age-standardized DALYs rate in 204 countries and territories in 2021; (5) All-age prevalence and DALYs, age-standardized prevalence and DALYs rate of five Socio-demographic Index (SDI) categories (low SDI, low-middle SDI, middle SDI, high-middle SDI and high SDI); (6) SDI values of 204 countries and territories in 2021.

### 2.2 National socioeconomic status

The SDI is a comprehensive measure of a geographical area’s development status based on three fundamental dimensions: total fertility rate (age < 25 years), mean education (age > 15 years), and lag-distributed income (LDI) per capita. The SDI is expressed on a scale of 0 to 1, strongly related to health outcomes. As a composite, a location with an SDI of 0 would have a theoretical minimum level of development relevant to health, while a location with an SDI of 1 would have a theoretical maximum level. The 2021 SDI values of 204 countries and territories were divided into five socioeconomic groups: high SDI (≥ 0.810), high-middle SDI (0.712 ≤ SDI < 0.810), middle SDI (0.619 ≤ SDI < 0.712), low-middle SDI (0.466 ≤ SDI < 0.619), and low SDI (< 0.466).

### 2.3 Statistical analyses

The data are expressed as mean estimates with 95% uncertainty intervals (UIs; the 25th and 975th estimates among the 1,000 draws). The GBD study used DisMod-MR 2.1, a Bayesian meta-regression tool, to estimate these metrics for each health loss condition. We compared the gender distinctions (males minus females) related to IOFBs among groups with different SDI using the Wilcoxon signed-rank test. Correlations between gender differences in IOFBs and SDI were estimated using linear regression. We used SPSS 26.0 Statistical software (IBM Corp) and Prism Software Version 10 (GraphPad Software) for statistical analysis. A value of *p* < 0.05 was considered statistically significant. *p* < 0.001 represented a very significant difference.

## 3 Results

### 3.1 Trends in global gender-specific burden of IOFBs

**Figure 1:**
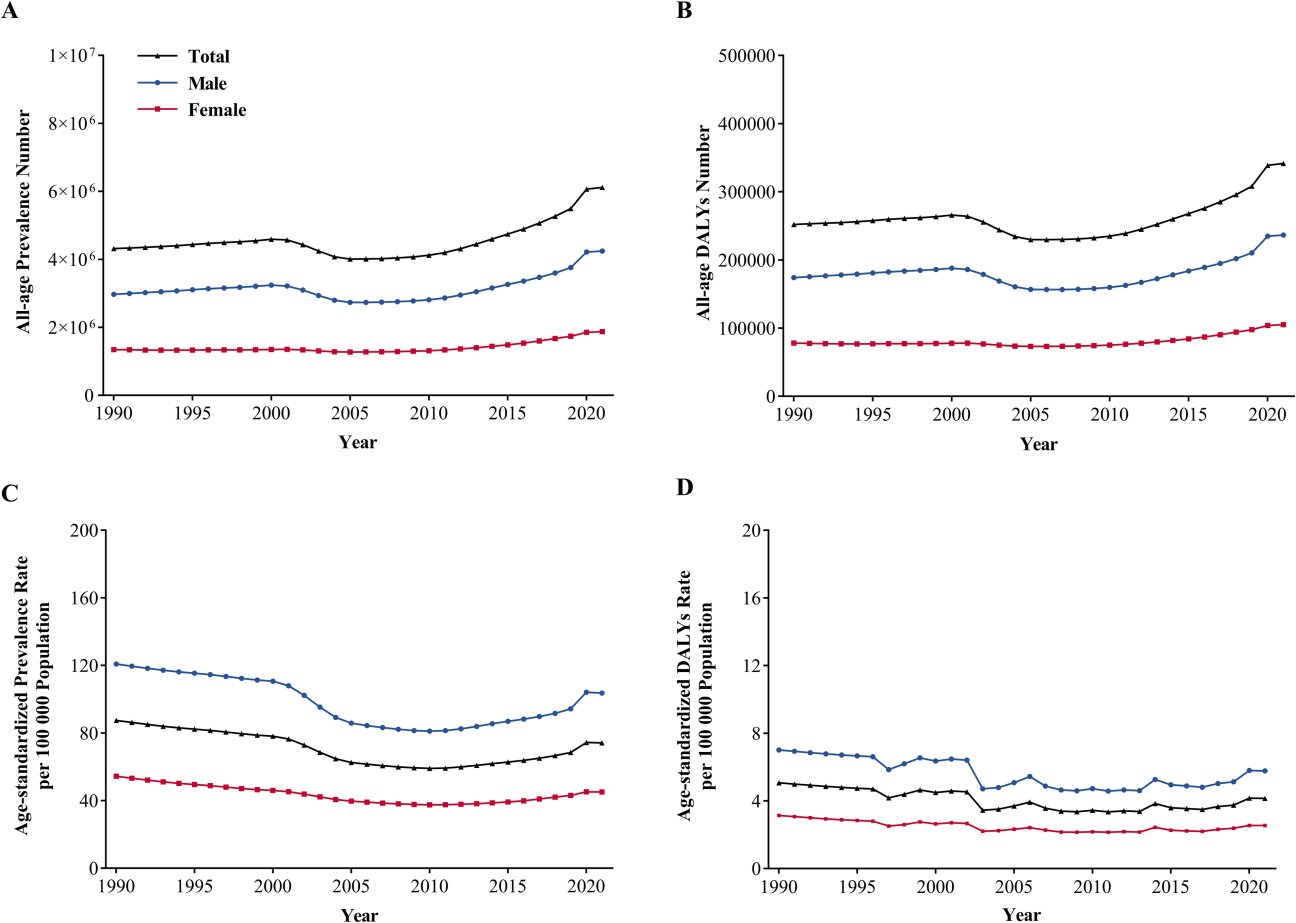
Global Gender-specific Burden of IOFBs by year. **(A)** All-age prevalence number from 1990 to 2021; **(B)** All-age DALYs number from 1990 to 2021; **(C)** Age-standardized prevalence rate from 1990 to 2021; **(D)** Age-standardized DALYs rate from 1990 to 2021. IOFBs, intraocular foreign bodies; DALYs, disability-adjusted life years.

The total all-age prevalence of IOFBs rose by 41.9%, from 4.3 (95% UI: 2.5–6.7) million in 1990 to 6.1 (95% UI: 3.6–9.2) million in 2021 (Figure 1A). The all-age prevalence number was 3.0 (95% UI: 1.6–4.7) million among men vs. 1.3 (95% UI: 0.8–2.0) million among women in 1990, and 4.2 (95% UI: 2.5–6.5) vs. 1.9 (95% UI: 1.1–2.7) million in 2021 (*p* < 0.001). The total all-age DALYs due to IOFBs increased by 35.5%, with 252.1 (95% UI: 132.3–441.5) thousand in 1990 vs. 341.6 (95% UI: 182.6–598.8) thousand in 2021 (Figure 1B). Gender disparity still exists in the all-age DALYs, with men showing a greater burden than women in 1990 (174.1 vs. 78.0 thousand, respectively) and 2021 (236.6 vs. 105.0 thousand, respectively) (*p* < 0.001). After controlling the effect of population size and age structure, the age-standardized prevalence rate decreased by 15.2% from 87.4 (95% UI: 51.7–133.2) per 100,000 population in 1990 to 74.1 (95% UI: 43.4–112.3) per 100,000 population in 2021 (Figure 1C). The age-standardized prevalence rates for males and females were 120.8 (95% UI: 70.0–186.1) vs. 54.4 (95% UI: 34.1–80.4) per 100,000 population in 1990 and 103.6 (95% UI: 60.2–158.2) vs. 45.1 (95% UI: 27.5–112.3) per 100,000 population in 2021 (*p* < 0.001). A similar decrease of 19.6% was found in the age-standardized DALYs rate from 5.1 (95% UI: 2.7–8.7) per 100,000 population in 1990 to 4.1 (95% UI: 2.2–7.3) per 100,000 population in 2021 (Figure 1D). Men had a higher age-standardized DALYs rate than women, with a change of 7.0 (95% UI: 3.8–12.3) vs. 3.1 (95% UI: 1.7–5.3) per 100,000 population in 1990 and 5.8 (95% UI: 3.0–10.2) vs. 2.5 (95% UI: 1.4–4.3) per 100,000 population in 2021 (*p* < 0.001). IOFBs had the most obvious gender differences compared with other eye diseases in GBD 2021 (Supplementary Table S1).

**Figure 2:**
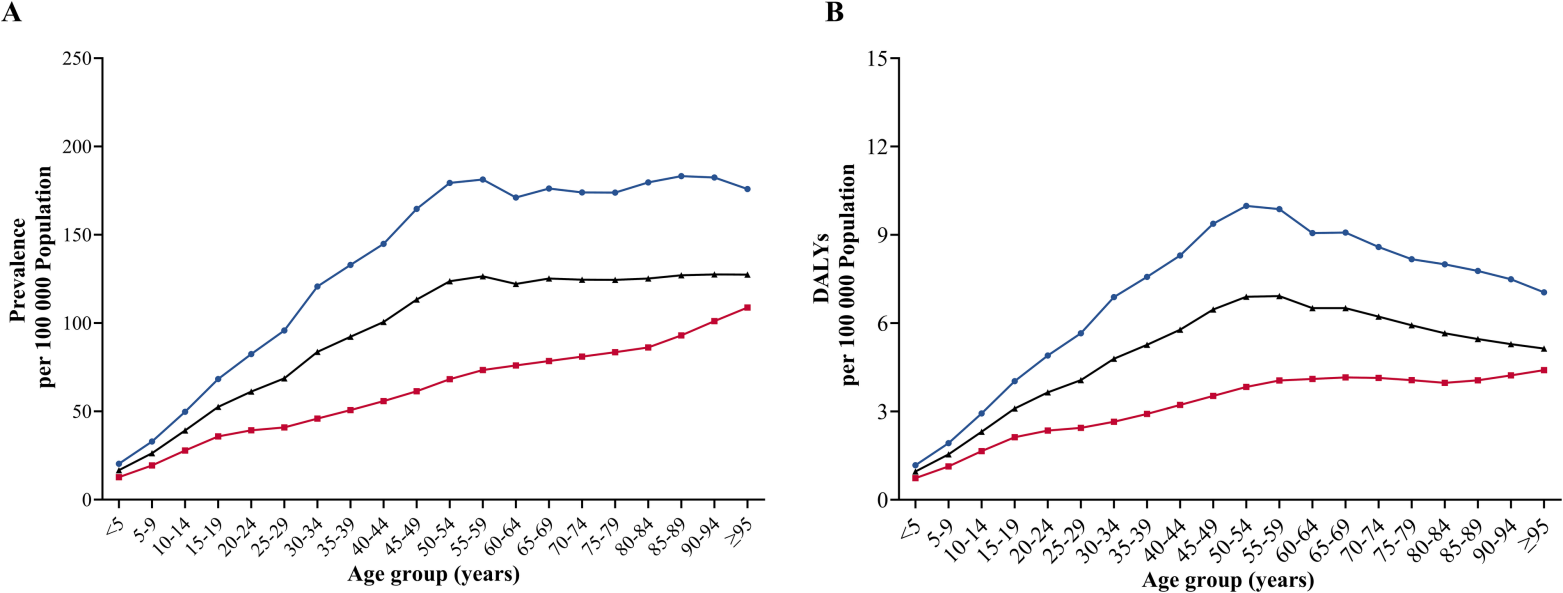
Global Gender-specific Burden of IOFBs by age. **(A)** Age-specific prevalence rate in 2021; **(B)** Age-specific DALYs rate in 2021. IOFBs, intraocular foreign bodies; DALYs, disability-adjusted life years.

Overall, the total and gender-specific burden of IOFBs reached a small peak among middle aged adults. Higher gender-specific prevalence and DALYs rates were discovered in men across different age groups. Gender disparities in the prevalence and DALY rates were more obvious among 30–44, 45–49, 50–54 and 55–59 years, with the most obvious difference in 45–49 years. The gender-specific prevalence rates of 45–49 group were 164.7 (95% UI: 86.0–309.8) per 100,000 men and 61.4 (95% UI: 35.1–109.4) per 100,000 women (Figure 2A). Similarly, the gender-specific DALYs rates of 45–49 group were 9.4 (95% UI: 4.3–19.6) per 100,000 men and 3.5 (95% UI: 1.8–6.8) per 100,000 women (Figure 2B).

### 3.2 Geographical distribution of IOFBs burden

**Figure 3:**
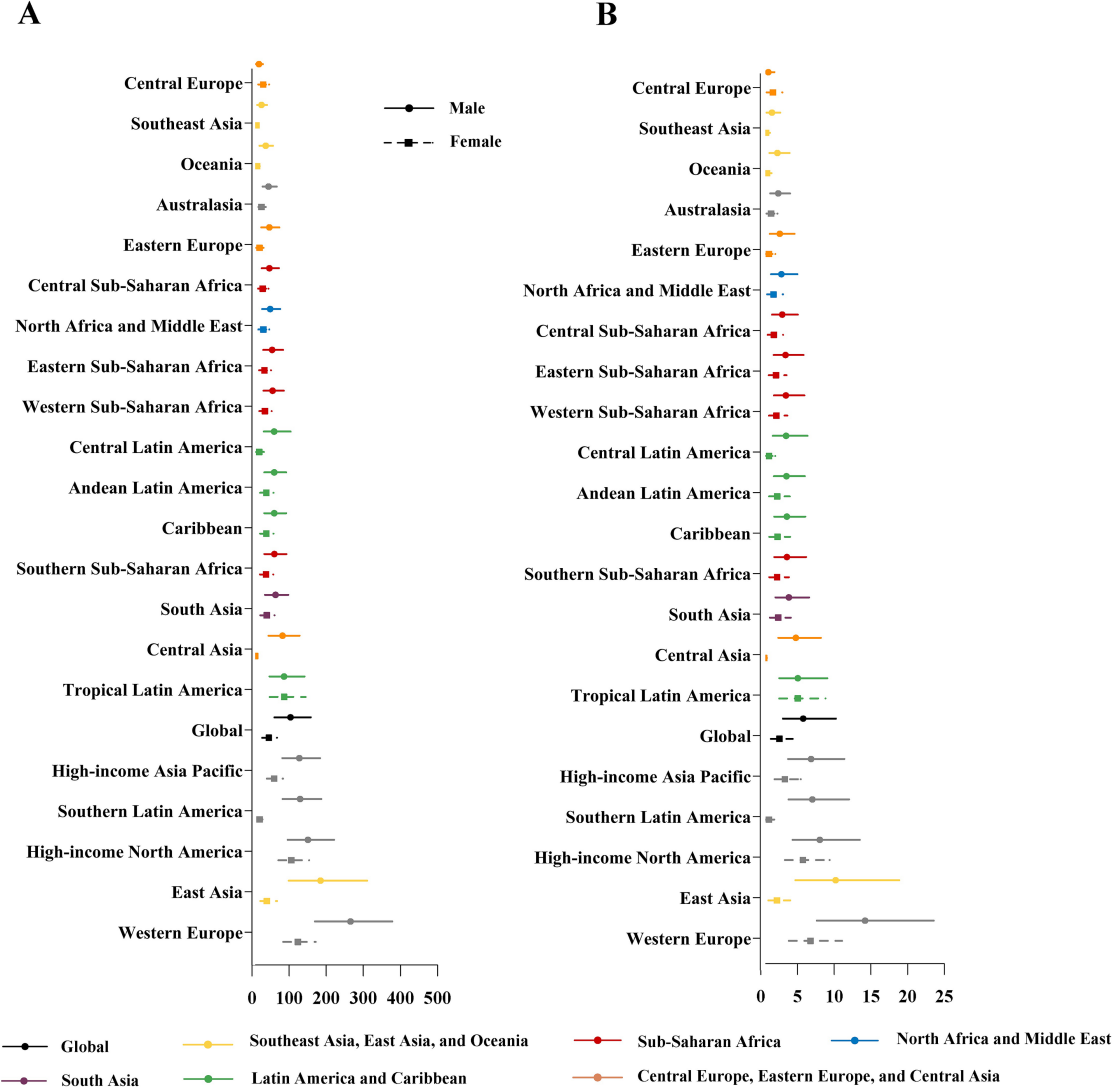
Gender-specific age-standardized prevalence **(A)** and DALYs rate **(B)** due to IOFBs by 21 GBD regions in 2021. DALYs, disability-adjusted life years; IOFBs, intraocular foreign bodies; GBD, Global Burden of Disease. Y-axis order is based on the level of the age-standardized prevalence and DALY rates in males.

The equations should be inserted in editable format from the equation editor. The age-standardized prevalence and DALYs rates of IOFBs in 2021 in different GBD regions are presented in Figures 3A,B, respectively. Supplementary Table S2 provides additional data on the all-age DALYs and the age-standardized DALYs rates of different GBD regions in 1990 and 2021. Significant gender disparities were observed in 21 GBD regions, and the IOFBs burden of men was greater than that of the women, based on the age-standardized prevalence and DALYs rates. Western Europe had the highest age-standardized prevalence rate for males (265.4, 95% UI:169.7–377.8) in 2021, followed by East Asia (184.6, 95% UI:98.8–310.3), High-income North America (150.7, 95% UI:96.3–221.0) and Southern Latin America (129.5, 95% UI:82.2–187.0). Similarly, Western Europe had the highest age-standardized DALYs rate for males (14.2, 95% UI:7.6–23.6) in 2021, followed by East Asia (10.2, 95% UI:4.7–18.8), High-income North America (8.0, 95% UI:4.3–13.5) and Southern Latin America (7.0, 95% UI:3.8–12.0).

**Figure 4:**
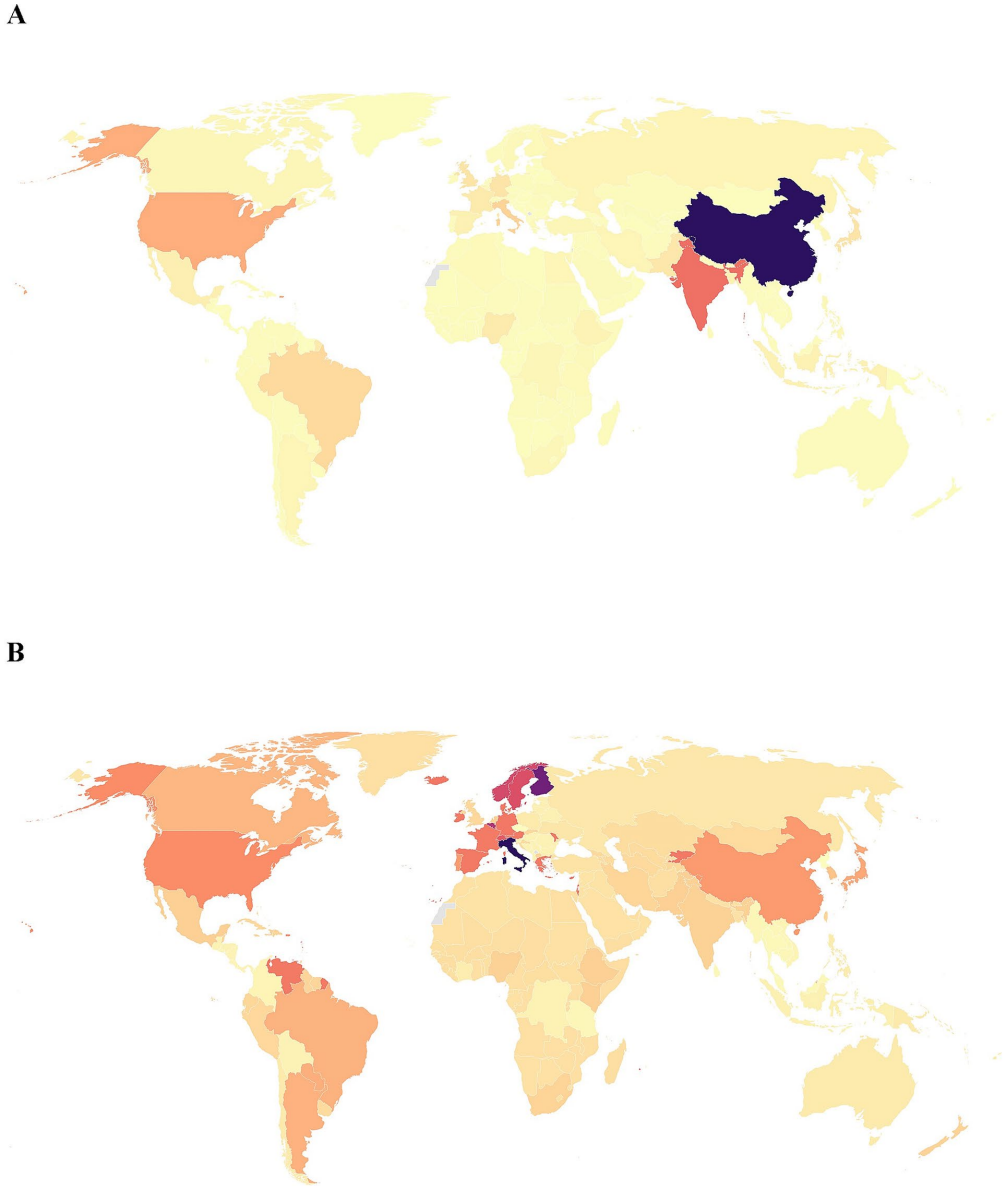
Maps of all-age DALYs number and age-standardized DALYs rate of IOFBs in different countries and territories in 2021. **(A)** All-age DALYs number; **(B)** Age-standardized DALYs rate per 100,000 population. DALYs, disability-adjusted life years; IOFBs, intraocular foreign bodies. The boundaries shown on the maps do not represent any opinions of authors. Gray areas indicate unavailabledata.

The global distribution of the burden of IOFBs by different countries and territories in 2021 was uneven (Figure 4). The all-age DALYs were highest in China [110062.8 (95% UI: 51569.5–200376.2) DALYs, male vs. female = 91410.4 (95% UI: 42500.2–165912.7) vs. 19652.4 (95% UI: 9391.3–35477.4) DALYs), followed by India, United States, Italy, Brazil and Japan (Figure 4A). The age-standardized DALYs rate was highest in Italy [20.5 (95% UI: 11.3–33.9)] per 100,000 population, male vs. female = 27.1 (95% UI: 14.6–45.3) vs. 13.9 (95% UI: 7.8–22.5) per 100,000 population), followed by Finland, Belgium, Norway, and Sweden (Figure 4B).

### 3.3 Socioeconomic disparity in IOFBs burden

**Figure 5:**
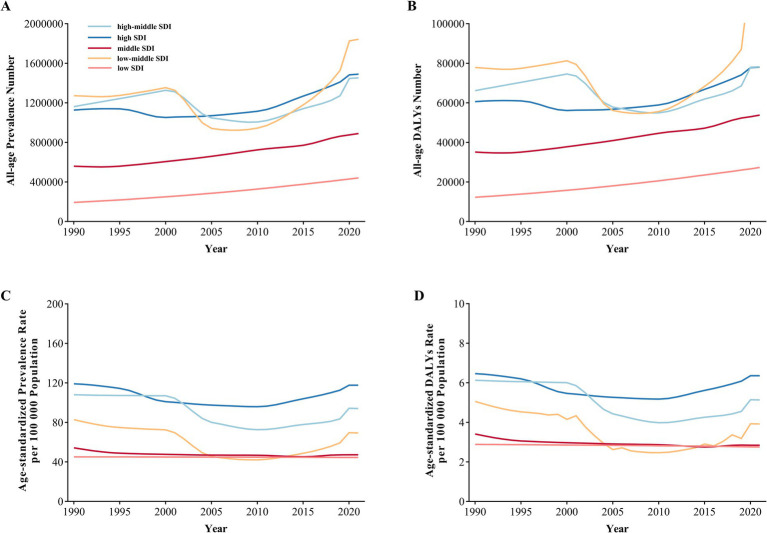
Global Burden of IOFBs by different SDI categories from 1990 to 2021. **(A)** All-age prevalence number; **(B)** All-age DALYs number; **(C)** Age-standardized prevalence rate; **(D)** Age-standardized DALYs rate. IOFBs, intraocular foreign bodies; DALYs, disability-adjusted life years; SDI, socio-demographic index.

The 2021 SDI values of 204 countries were divided into five socioeconomic groups: high SDI, high-middle SDI, middle SDI, low-middle SDI, and low SDI. SDI data were available for 204 countries and territories, including high SDI countries (*n* = 42), high-middle SDI countries (*n* = 45), middle SDI countries (*n* = 41), low-middle SDI countries (*n* = 42), and low SDI countries (*n* = 34). The all-age prevalence and DALYs numbers of five SDI categories all showed an overall upward trend since 1990 to 2021 (Figures 5A,B). The low-middle SDI group had the highest all-age prevalence and DALY numbers in 2021. After controlling the effect of population size and age structure, the high SDI group indicated the highest age-standardized prevalence and DALY rates in 2021 (Figures 5C,D).

**Figure 6:**
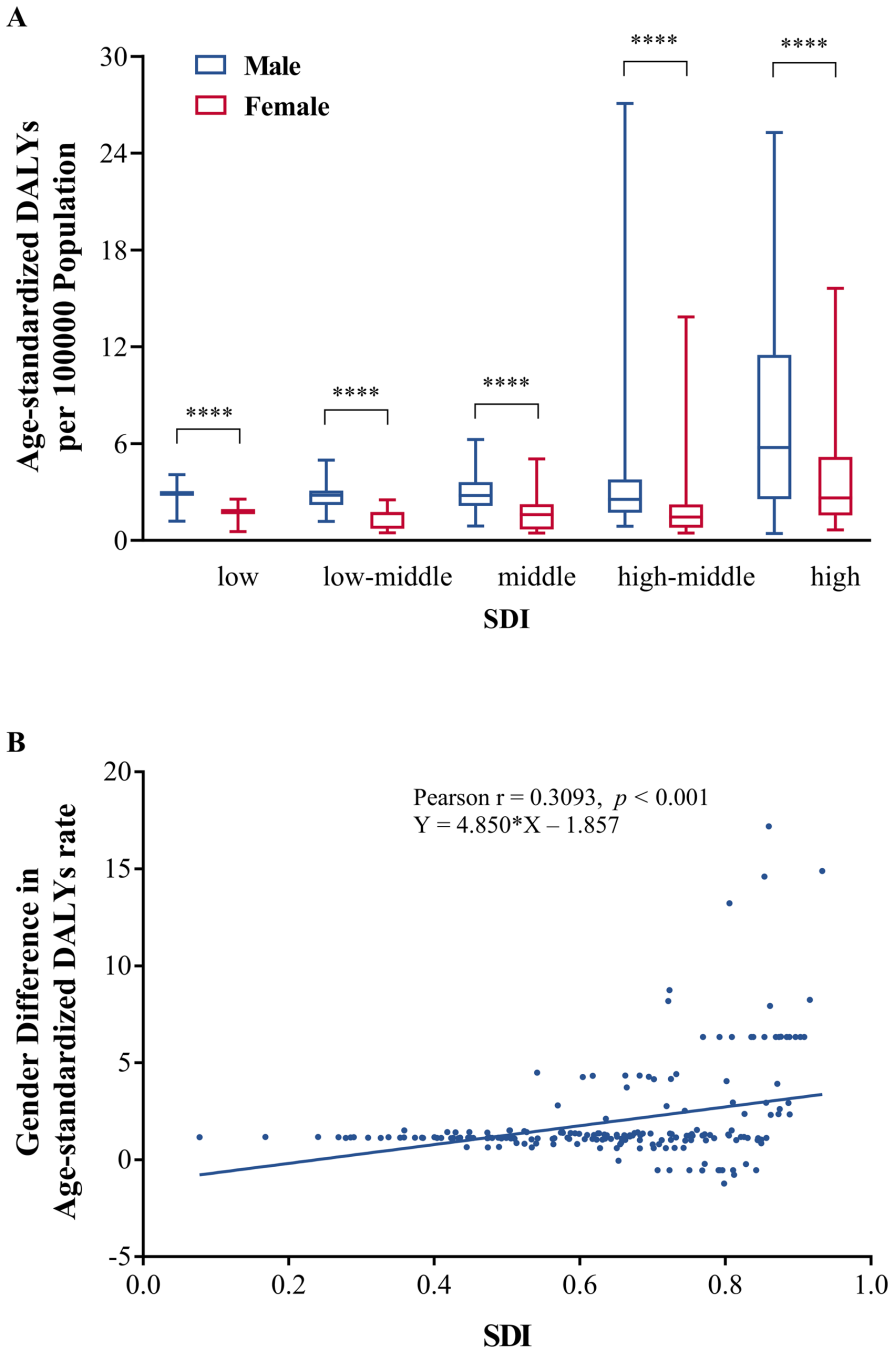
Relationship between age-standardized DALYs rate of IOFBs and SDI. **(A)** Age-standardized DALYs rate due to IOFBs of males and females among different SDI regions; **(B)** Association between gender difference (male minus female) in age-standardized DALY rates of IOFBs and SDI. DALYs, disability-adjusted life years; IOFBs, intraocular foreign bodies; SDI, socio-demographic index. ^****^p < 0.0001.

In Figure 6A, Wilcoxon signed-rank test indicated that the age-standardized DALYs rates in 2021 of males were higher than the rates of females in low (medians = 2.9 vs. 1.7), low-middle (2.8 vs. 1.7), middle (2.8 vs. 1.6), high-middle (2.6 vs. 1.5), and high-SDI countries (5.8 vs. 2.6) (all *p* < 0.001). Pearson’s correlations (r = 0.3093, *p* < 0.001) and linear regression analysis (Y = 4.850*X − 1.857) revealed that gender differences (male minus female) in the age-standardized DALYs rate of 204 countries and territories were positively associated with the SDI (Figure 6B).

### 3.4 Global burden due to various eye diseases in the GBD 2021

In addition to IOFBs, we found that other eye diseases also created major global burdens in the GBD 2021 study. We compared the all-age DALYs and age-standardized DALYs rates due to various eye diseases by gender in 1990 and 2021 (Supplementary Table S1). The IOFBs ranked seventh in terms of the total all-age DALYs and the age-standardized DALYs rates in 1990 and 2021, behind near vision loss, refraction disorders, cataract, other vision loss, glaucoma, and age-related macular degeneration. Fortunately, the increase in the all-age DALYs of IOFBs between 1990 and 2021 was lowest among these seven eye diseases. Furthermore, the age-standardized DALYs rates of six eye diseases decreased from 1990 to 2021 except for near vision loss. The IOFBs had the greatest gender-related differences compared with other eye disorders.

## 4 Discussion

Injuries due to IOFBs remain an important cause of visual loss in developing and developed countries. This study demonstrates that the burden of IOFBs has been considerable burden in middle-aged and older adult men over the past few decades. Men suffered more burden of IOFBs than women by different year, age group, region, country and SDI. The distribution of burden of IOFBs is uneven among regions and countries, higher in countries of Western Europe, East Asia, High-income North America and Southern Latin America. After controlling the effect of population size and age structure, countries with higher SDI had larger gender difference in age-standardized DALYs rate. This reminds us that we cannot ignore the IOFBs burden of all countries.

The global all-age prevalence and DALYs showed an upward trend from 1990 to 2021 with a small peak in 2000, which was probably caused by a rapidly aging and growing population, increasing life expectancy, rising incidence of work-related injuries and accidents, and lack of eye protection. The epidemiology of IOFB injuries has been studied in many countries and territories, such as China ^[6]^, Ireland (20), and Hong Kong (21). Similar increases in the prevalence and DALYs were observable in several common ocular diseases during the same period, including glaucoma, cataract, diabetic retinopathy, and age-related macular degeneration (22–25). The age-standardized prevalence and DALYs rates of IOFBs declined slightly between 1990 and 2021. Similarly, the incidence of OGIs in the US decreased from 5.88 per 1,000,000 population in 2006 to 3.92 per 1,000,000 population in 2014 (26). This trend might be attributed to the decline in industrialization and improvements in occupational-safety awareness. The advancement of medical imaging technology and ophthalmic microsurgery technology might also contribute to the declining pattern.

In the GBD 2021, men accounted for the majority of the burden of IOFBs, which was similar to results of previous studies (8). Unlike the IOFBs, women had a larger disease burden due to age-related macular degeneration, cataract, and diabetic retinopathy than men in the GBD study (22–24, 27). Although the overall trend in the burden of IOFBs fluctuated from 1990 to 2021, males consistently showed a substantial burden compared to females in our study. The male/female ratio of eye injury varied from 1.8:1 to 8.0:1 (3, 26, 28, 29). A literature review conducted by Loporchio et al. reported that young men made up 92–100% of the patients with IOFBs (30). There are several reasons for these gender differences. First, most IOFB injuries occurred during work (54–72%) (30). IOFBs have always been serious complications of work-related injuries, caused by hammering, chiseling, and machine-related accidents. These jobs have been almost exclusively male jobs; therefore, men suffered a higher incidence of traumatic occupational accidents. In addition to occupational exposure, gender disparities in IOFB burden may also be influenced by social and cultural factors. Traditional gender roles often assign men to high-risk jobs and may discourage timely medical care. These norms, combined with differences in healthcare access and economic pressures, may contribute to the higher burden observed in men across various regions. Second, the proper use of safety glasses and other protective equipment can prevent most of these injuries (31). Although the awareness of eye protection has increased in recent decades, the rate of eye protection is low during high-risk work procedures in the workplace, ranging from 0.77 to 6% (9, 32). Third, Croce et al. demonstrated that men tended to have more infectious complications than women among trauma patients, particularly in young patients (33). The gender differences might be related to high testosterone and low estradiol levels (34).

We found that the burden of IOFBs was concentrated in middle-aged and older adult men, especially among 30–44, 45–49, 50–54 and 55–59 years. Men are more likely to work outdoors and, therefore, injure their eyes. The average age when people have eye injuries is 29–42 years old (6, 9, 32, 35). Low income and educational level make it difficult for the patients to understand the need to undergo eye operations. In a retrospective study of 812 cases with OGIs (14), 17% of workers did not have a medical evaluation within 12 h of their OGIs, and 10% did not have an evaluation within 24 h of their OGIs. Eye injuries can result in lost wages and earning potential for individuals, and an economic loss for society. IOFBs often cause mechanical injuries and pathological changes, such as ocular siderosis, chalcosis, iridocyclitis, endophthalmitis, vitreous hemorrhage, ocular hypotony, and retinal detachment. Hence, an eye injury could affect patients’ vision for many years in their remaining lives. Except for young and older men, children’s eyeballs are often penetrated by toy guns, plant spines, and writing tools, and these injuries require timely medical care to ensure the best possible outcomes (36).

In the US, the costs of OGIs from 2006 to 2014 accounted for $793 million (8.3%) in total ocular-trauma charge (26). Hospitalization and the occurrence of disability increase with the prevalence of OGIs. Although the direct (long-term treatment, surgery, and hospitalization) and indirect (disability, loss of labor productivity, and reduced quality of life) costs of eye injuries could reach hundreds of millions of dollars, little has been spent on eye-injury research (15). Compared with common eye diseases, there were fewer clinical reports and financial investment on IOFBs. A study (37) conducted in India reported the average cost of OGIs exceeded the monthly income of 84.8% of patients, suggesting a significant financial strain in low-income settings. In Australia, OGIs accounted for only 2% of ocular trauma cases but represented 44% of the total treatment costs, indicating a disproportionately high financial burden relative to case numbers (38). Similarly, in the United Kingdom, a randomized controlled trial (27) reported that the average cost of OGI treatment was £5,526 per patient. These findings underscore the substantial and varied economic impact of OGIs across different healthcare systems. However, comprehensive data from low-and middle-income countries remain limited, highlighting the need for broader international research on the economic consequences of OGIs.

In our study, the burden of IOFBs varied widely among countries and territories. The all-age DALYs of IOFBs in 2021 were greatest in China, India and United States. The age-standardized DALYs rate was concentrated in Western Europe. There are many factories and workers in these countries, resulting in unavoidable occupational OGIs. Although China has undergone rapid socioeconomic development, its industrial structure is dominated by the manufacturing industry. The large burden of IOFBs in China might be related to the large workforce and inadequate workplace protection for young male workers (6). Western European countries are renowned for advanced manufacturing and industrial sectors. Workers in these industries are frequently exposed to high-speed tools and flying particles such as metal debris, increasing the likelihood of IOFB incidents. The prevalence of industrial activities, technical jobs, and mechanization in these countries inherently increases the occupational risk of IOFBs.

Among different GBD regions, the burden of IOFBs was higher in Western Europe, East Asia, High-income North America and Southern Latin America. Countries with high and high-middle SDI had higher burden and more obvious gender difference. There are several possible reasons for our findings. Firstly, high-SDI regions have higher levels of industrialization with more high-risk occupations, such as machining, construction, and metalworking. These high-risk occupations increase the risk of IOFBs. Besides industrialization, differences in healthcare systems and safety regulation enforcement may also affect gender disparities in IOFB burden. High-SDI countries often have better reporting, but safety standards may not be fully enforced, especially in small businesses, putting male workers at risk. Moreover, the people in high-SDI regions may engage more frequently in high-risk recreational activities, such as DIY home improvement or crafting. High-SDI regions often have more technology-intensive industries where the use of complex tools and equipment is more frequent, exposing workers to higher risks of IOFB. Secondly, high-SDI regions have well-developed healthcare systems and stronger public health awareness, making it easier to identify and record IOFB cases. In contrast, in lower SDI regions, some minor IOFB cases may remain undiagnosed or unreported. They might not afford the healthcare costs or routine eye follow-up, leading to the lack of statistics. Thirdly, although high-SDI regions may provide better occupational safety training and protective equipment, these measures might not be fully implemented, especially in small businesses or informal employment sectors. Lower SDI regions might have fewer people engaged in high-risk occupations, leading to a lower incidence of IOFB. The data may be influenced by statistical bias. For instance, cases in high SDI regions are more likely to be included in research or statistics, whereas lower SDI regions might lack comprehensive monitoring and documentation. Pearson’s correlation coefficient of r = 0.3093 suggests a moderate relationship between SDI and gender differences in IOFBs burden. However, this relationship may be influenced by confounding factors such as healthcare accessibility indicators, GDP, and education. Further analysis using more granular individual-level data are needed to further disentangle the effects of healthcare access, occupational exposure, and socioeconomic status.

However, areas with a lower socioeconomic status tended to have more ocular injuries (39, 40), which might be associated with poor eye-protection equipment and inadequate health care in poorer regions. Further analysis could focus on specific environmental factors, such as industry structure and occupational distribution. Poverty was associated with eye disorders (41), and their burden was always clustered in countries with lower socioeconomic development. The positive correlation of our study is usually the result of multiple factors and requires further study that considers the characteristics of specific regions and populations.

We conducted comprehensive epidemiologic evaluation on gender and socioeconomic disparities of IOFBs during a 30-year period, using the latest data. Our findings are informative for implementing measures to prevent eye injuries in males by country, region and socioeconomic status. We have also reported the global burden of IOFBs and other ocular diseases to emphasize the necessity for preventing and treating them. The observed gender disparities in IOFBs may result from a complex interplay of occupational risk, societal expectations, and disparities in healthcare access. To effectively reduce the burden of IOFBs, particularly among high-risk populations such as working-age men in industrial and agricultural sectors, targeted preventive strategies are essential. These include the mandatory use of certified protective eyewear in high-risk occupations, strict enforcement of workplace safety regulations, regular safety training programs, improved access to eye health services for women in underserved areas. In the United Arab Emirates, despite 85% of workers performing high-risk tasks, none consistently used eye protection, underscoring the need for stronger educational and enforcement interventions (42). A cohort in Shanghai demonstrated that despite widespread provision of protective eyewear, up to 20% of workers still suffered eye injuries due to poorly fitting or misused equipment (43). Moreover, integrating eye protection protocols into standard occupational health policies, especially in low-SDI countries, could significantly reduce the burden of IOFBs.

This study has some limitations. First, non-magnetic IOFBs include metallic and nonmetallic foreign bodies, which cause severe inflammatory reactions (30). Treatments and complications of magnetic and non-magnetic IOFBs are different; thus, they should be distinguished and investigated in future studies. Second, our analyses are affected by the limitations of the GBD 2021 study. The accuracy of our findings was associated with the data sources and statistical methods of typical literature included in the GBD 2021 study. The publication bias and heterogeneity cannot be ruled out. This study may be affected by potential limitations such as underreporting, regional variability in diagnostic practices, and the reliance on estimates instead of direct empirical measurements. The aggregated nature of GBD data may conceal important sub-national or community-level disparities and overlook contextual factors that influence the burden of IOFBs. In low-SDI regions, underreporting due to limited access to healthcare services and inadequate surveillance systems may result in underestimated IOFB burdens. Moreover, estimates from countries with insufficient health data systems rely more heavily on modeling assumptions, which may affect their accuracy. Furthermore, IOFBs were classified in the GBD 2021 study as unintentional injuries, not as eye diseases. Yet, many prognostic factors affect the final visual acuity of patients with IOFBs, including the volume and nature of foreign bodies, the injury site, and the presence of serious damage or infection (21, 44). On the whole, our study identifies correlations between the burden of IOFBs, gender and SDI. However, it is important to note that these relationships are correlational and do not establish direct causation. Visual impairment caused by IOFBs should be examined in further studies.

## 5 Conclusion

The all-age prevalence and DALYs due to IOFBs increased over the past few decades, while the age-standardized prevalence and DALY rates showed a downward trend. Globally, IOFBs remains a significant risk factor for vision loss, especially among middle-aged and older adult men. The burden of IOFBs had distinct geographic patterns: gender differences were more evident in areas with higher SDI. As technology for medical imaging examination and surgery improve, policies and strategies for eye protection and occupational safety should be emphasized.

## Data Availability

The datasets presented in this study can be found in online repositories. The names of the repository/repositories and accession number(s) can be found in the article/Supplementary material.

## Footnotes

^1^ http://ghdx.healthdata.org/gbd-data-tool

## Supplementary material

SUPPLEMENTARY TABLE S1 All-age DALYs and age-standardized DALYs rates due to various eye diseases in 1990 and 2021 with percent change. DALYs, disability-adjusted life years.

SUPPLEMENTARY TABLE S2 All-age DALYs and age-standardized DALYs rate of IOFBs by GBD Regions in 1990 and 2021. DALYs, disability-adjusted life years; IOFBs, intraocular foreign bodies; GBD, Global Burden of Disease.
