# Supplementary material for: Global, regional and national patterns and gender disparity of intraocular foreign bodies from 1990 to 2021
Source: Front Public Health. 2025 Jun 25;13:1620358. doi: 10.3389/fpubh.2025.1620358 (PMC12238015; doi:10.3389/fpubh.2025.1620358)
Supplement: SUPPLEMENTARY TABLE S1 — All-age DALYs and age-standardized DALYs rates due to various eye diseases in 1990 and 2021 with percent change. DALYs, disability-adjusted life years. [file Table_1.doc]

**Supplementary Table 1.**

**All-age DALYs and age-standardized DALYs rates due to various eye diseases in 1990 and 2021 with percent change.**

| **Eye diseases** | **All-age DALYs (thousands)** | | | | | | **Change (%)** | **Age-standardized DALYs rate (per 100 000 population)** | | | | | | **Change (%)** |
| --- | --- | --- | --- | --- | --- | --- | --- | --- | --- | --- | --- | --- | --- | --- |
|  | **1990** | | | **2021** | | |  | **1990** | | | **2021** | | |  |
|  | **Male** | **Female** | **Total** | **Male** | **Female** | **Total** |  | **Male** | **Female** | **Total** | **Male** | **Female** | **Total** |  |
| **IOFBs** | **174.1** | **78.0** | **252.1** | **236.6** | **105.0** | **341.6** | **35.5** | **7.0** | **3.1** | **5.1** | **5.8** | **2.5** | **4.1** | **-19.6** |
| Age-related macular degeneration | 119.1 | 183.8 | 302.9 | 232.6 | 345.4 | 578.0 | 90.8 | 7.4 | 9.0 | 8.4 | 6.0 | 7.4 | 6.8 | -19.0 |
| Glaucoma | 247.4 | 220.2 | 467.6 | 397.2 | 362.7 | 759.9 | 62.5 | 16.8 | 11.0 | 13.4 | 10.8 | 7.7 | 9.1 | -32.1 |
| Other vision loss | 832.1 | 948.9 | 1781.0 | 1384.5 | 1619.2 | 3003.7 | 68.6 | 42.6 | 43.0 | 42.9 | 34.6 | 36.0 | 35.3 | -17.7 |
| Cataract | 1467.3 | 1949.3 | 3416.6 | 2711.2 | 3842.6 | 6553.8 | 91.8 | 88.7 | 93.6 | 91.1 | 69.9 | 83.0 | 77.0 | -15.5 |
| Refraction disorders | 1893.3 | 2135.8 | 4029.1 | 3069.3 | 3549.3 | 6618.6 | 64.3 | 85.7 | 90.5 | 88.0 | 75.5 | 82.6 | 79.1 | -10.1 |
| Near vision loss | 1875.9 | 2440.2 | 4316.1 | 5201.9 | 6448.1 | 11650.0 | 169.9 | 88.2 | 107.8 | 98.3 | 123.8 | 146.8 | 135.5 | 37.8 |

DALYs, disability-adjusted life years; IOFBs, intraocular foreign bodies. Change (%) means percentage change in total all-age DALYs and age-standardized DALYs. rates between 1990 and 2021.
