# Supplementary material for: Global, regional and national patterns and gender disparity of intraocular foreign bodies from 1990 to 2021
Source: Front Public Health. 2025 Jun 25;13:1620358. doi: 10.3389/fpubh.2025.1620358 (PMC12238015; doi:10.3389/fpubh.2025.1620358)
Supplement: SUPPLEMENTARY TABLE S2 — All-age DALYs and age-standardized DALYs rate of IOFBs by GBD Regions in 1990 and 2021. DALYs, disability-adjusted life years; IOFBs, intraocular foreign bodies; GBD, Global Burden of Disease. [file Table_2.doc]

**Supplementary Table 2.**

**All-age DALYs and age-standardized DALYs rate of IOFBs by GBD Regions in 1990 and 2021.**

| **GBD 2021 Super Region** |  | **All-age DALYs (thousands)** | | | | | | | | |  |  | **Age-standardized DALYs rate**  **(per 100 000 population)** | | | | | | |
| --- | --- | --- | --- | --- | --- | --- | --- | --- | --- | --- | --- | --- | --- | --- | --- | --- | --- | --- | --- |
|  |  | | **1990** | |  |  |  | **2021** | |  |  |  | | **1990** |  |  |  | **2021** |  |
|  | **Male** | | **Female** | **Total** | |  | **Male** | **Female** | **Total** | |  | **Male** | **Female** | | **Total** |  | **Male** | **Female** | **Total** |
| **Global** | 294.4 | | 126.7 | 421.2 | |  | 399.4 | 191.0 | 590.4 | |  | 7.0 | 3.1 | | 5.1 |  | 5.8 | 2.5 | 4.1 |
| **Latin America and Caribbean** | 18.5 | | 11.0 | 29.6 | |  | 31.4 | 19.6 | 51.0 | |  | 7.4 | 6.1 | | 6.7 |  | 4.1 | 2.8 | 3.4 |
| Tropical Latin America | 8.9 | | 5.4 | 14.3 | |  | 15.1 | 9.6 | 24.7 | |  | 12.9 | 12.6 | | 12.8 |  | 5.0 | 5.0 | 5.0 |
| Central Latin America | 7.2 | | 4.3 | 11.5 | |  | 12.4 | 7.7 | 20.1 | |  | 3.6 | 1.2 | | 2.4 |  | 3.4 | 1.1 | 2.2 |
| Andean Latin America | 1.2 | | 0.7 | 1.9 | |  | 2.2 | 1.2 | 3.5 | |  | 3.8 | 2.5 | | 3.1 |  | 3.5 | 2.2 | 2.9 |
| Caribbean | 1.2 | | 0.7 | 1.9 | |  | 1.7 | 1.0 | 2.7 | |  | 3.7 | 2.3 | | 3.0 |  | 3.5 | 2.3 | 2.9 |
| **Southeast Asia, East Asia, and Oceania** | 147.8 | | 42.1 | 189.9 | |  | 163.2 | 50.2 | 213.4 | |  | 8.9 | 2.0 | | 5.5 |  | 7.6 | 1.7 | 4.7 |
| Southeast Asia | 13.2 | | 4.7 | 18.0 | |  | 22.4 | 7.9 | 30.3 | |  | 1.5 | 0.7 | | 1.1 |  | 1.5 | 0.7 | 1.1 |
| East Asia | 134.4 | | 37.3 | 171.8 | |  | 140.5 | 42.2 | 182.7 | |  | 11.3 | 2.4 | | 7.0 |  | 10.2 | 2.2 | 6.3 |
| Oceania | 0.1 | | 0.0 | 0.2 | |  | 0.3 | 0.1 | 0.4 | |  | 2.3 | 0.8 | | 1.6 |  | 2.3 | 0.8 | 1.6 |
| **North Africa and Middle East** | 12.0 | | 6.3 | 18.3 | |  | 23.8 | 12.2 | 36.0 | |  | 3.1 | 1.9 | | 2.5 |  | 2.8 | 1.7 | 2.3 |
| **South Asia** | 50.0 | | 25.5 | 75.5 | |  | 88.0 | 47.7 | 135.7 | |  | 4.0 | 2.5 | | 3.3 |  | 3.8 | 2.4 | 3.1 |
| **Sub-Saharan Africa** | 16.7 | | 9.6 | 26.3 | |  | 36.9 | 22.0 | 58.9 | |  | 3.5 | 2.1 | | 2.8 |  | 3.8 | 2.4 | 3.1 |
| Southern Sub-Saharan Africa | 2.2 | | 1.4 | 3.5 | |  | 3.7 | 2.3 | 6.0 | |  | 3.7 | 2.3 | | 3.0 |  | 3.6 | 2.2 | 2.9 |
| Eastern Sub-Saharan Africa | 5.9 | | 3.4 | 9.3 | |  | 13.4 | 7.7 | 21.1 | |  | 3.5 | 2.1 | | 2.8 |  | 3.4 | 2.1 | 2.7 |
| Central Sub-Saharan Africa | 1.3 | | 0.8 | 2.1 | |  | 3.3 | 1.8 | 5.1 | |  | 3.0 | 1.8 | | 2.4 |  | 2.9 | 1.8 | 2.3 |
| Western Sub-Saharan Africa | 7.3 | | 4.1 | 11.3 | |  | 16.6 | 10.1 | 26.7 | |  | 3.6 | 2.2 | | 2.9 |  | 3.4 | 2.1 | 2.7 |
| **Central / East Europe and Central Asia** | 16.2 | | 8.5 | 24.7 | |  | 16.5 | 8.8 | 25.3 | |  | 2.5 | 1.2 | | 1.9 |  | 2.7 | 1.1 | 1.9 |
| Central Europe | 4.1 | | 2.3 | 6.4 | |  | 4.0 | 2.3 | 6.3 | |  | 1.1 | 1.7 | | 1.4 |  | 1.0 | 1.6 | 1.3 |
| Eastern Europe | 9.8 | | 5.3 | 15.0 | |  | 9.2 | 5.2 | 14.4 | |  | 2.7 | 1.2 | | 1.9 |  | 2.6 | 1.1 | 1.8 |
| Central Asia | 2.3 | | 0.9 | 3.2 | |  | 3.4 | 1.3 | 4.6 | |  | 4.9 | 0.5 | | 2.6 |  | 4.8 | 0.5 | 2.6 |
| **High Income** | 33.2 | | 23.7 | 56.9 | |  | 39.4 | 30.7 | 70.1 | |  | 11.0 | 6.1 | | 8.5 |  | 10.1 | 5.3 | 7.7 |
| High-income Asia Pacific | 8.1 | | 5.3 | 13.4 | |  | 9.5 | 6.3 | 15.8 | |  | 7.0 | 3.4 | | 5.2 |  | 6.8 | 3.3 | 5.1 |
| Western Europe | 9.7 | | 6.2 | 15.9 | |  | 11.3 | 7.2 | 18.4 | |  | 15.9 | 8.6 | | 12.2 |  | 14.2 | 6.8 | 10.5 |
| Australasia | 0.6 | | 0.4 | 1.0 | |  | 0.9 | 0.6 | 1.5 | |  | 2.5 | 1.6 | | 2.1 |  | 2.4 | 1.4 | 1.9 |
| High-income North America | 13.8 | | 11.1 | 24.9 | |  | 16.5 | 15.6 | 32.0 | |  | 8.1 | 5.5 | | 6.8 |  | 8.0 | 5.7 | 6.9 |
| Southern Latin America | 0.9 | | 0.7 | 1.7 | |  | 1.3 | 1.0 | 2.3 | |  | 7.2 | 1.1 | | 4.1 |  | 7.0 | 1.1 | 4.0 |

GBD, global burden of disease; DALYs, disability-adjusted life-years; IOFBs, intraocular foreign bodies.
